# Editorial: Bilateral Vestibulopathy - Current Knowledge and Future Directions to Improve its Diagnosis and Treatment

**DOI:** 10.3389/fneur.2018.00762

**Published:** 2018-09-11

**Authors:** Bryan K. Ward, Alexander A. Tarnutzer

**Affiliations:** ^1^Department of Otolaryngology-Head and Neck Surgery, Johns Hopkins University School of Medicine, Baltimore, MD, United States; ^2^Department of Neurology, University Hospital Zurich and University of Zurich, Zurich, Switzerland

**Keywords:** bilateral vestibulopathy, labyrinth, oscillopsia, Walter Dandy, dizziness

Bilateral vestibulopathy has had several names, paralleling growing numbers of publications and interest in its pathophysiology and treatment. The condition was formerly Dandy syndrome, eponymously associated with neurosurgeon Walter Dandy who worked at the Johns Hopkins Hospital from 1918 to 1946. Having developed expertise in vestibular schwannoma surgery, Dandy became interested in vestibular nerve section to treat Meniere's disease. He achieved early success at controlling vertigo in patients with Meniere's disease after unilateral sectioning of the vestibular nerve, and then began performing bilateral vestibular nerve section. The first descriptions of the consequences of this surgery were in 1936 by neurologist Frank Ford and neuro-ophthalmologist Frank Walsh, who both worked with Dandy at the Johns Hopkins Hospital (1). They noted in a patient: “Objects seemed to move before his eyes unless his head was kept perfectly still.” Dandy later synthesized these cases and reported them himself in 1941, leading to the term Dandy syndrome (2).

Although we understand the implications of cutting both vestibular nerves today, at the time, many clinicians had a poor knowledge of the role of the vestibular system. A popular notion held that the vestibular system was vestigial. Prominent English physician Edmund Hobhouse noted in a 1924 Lancet editorial: “We are driven to the somewhat painful conclusion that in the semicircular canals man possesses a beautiful and complex mechanism which has been superseded by higher development, and whose only positive function now is to produce some of the most disabling and distressing symptoms which the human body can experience; moreover, this mechanism is so bound up with the organ of hearing that it is impossible to remove it without inflicting the penalty of deafness (3).” If vestibular testing and disease produces vertigo and nausea in those with intact labyrinthine function, and no symptoms in those without, Hobhouse argued, then the labyrinth is superfluous. Since airplane pilots could be led asunder by normal vestibular perceptions, the United States military even expressed interest in bilateral vestibulopathy to make better pilots (4). Dandy began performing vestibular nerve section for Meniere's disease in 1924, the same year as Hobhouse's editorial (5), later expanding to sectioning both vestibular nerves (6). Dandy comments in his 1934 series: “One is amazed that almost no symptoms are induced by the abrupt loss of both semicircular canals in man.” and provocatively states “It would be interesting indeed, to know whether this patient would be subject to seasickness.” Ford and Walsh' 1938 article describing Dandy's patients was in response to the popular belief that an intact vestibular system could only do harm.

This research topic highlights the significant progress our field has made since Ford and Walsh's first descriptions of Dandy syndrome (see Hain et al. for comprehensive review). Surgery is no longer a common cause of bilateral vestibulopathy, and the etiology of bilateral vestibulopathy is more varied than once believed (Kattah). Fortunately, idiopathic cases of bilateral vestibulopathy are becoming less common. Authors in this issue have identified new causes such as amiodarone (Gürkov) and environmental toxicities like a type of military jet fuel (Fife et al.). Others highlighted here include an accumulation of iron called superficial siderosis (Lee et al.), as well as central vestibular lesions that can mimic peripheral ones (Chen and Halmagyi).

New diagnostic tests like video head impulse testing (vHIT) and vestibular-evoked myogenic potentials (VEMPs, see Rosengren et al. for review) allow us to identify patterns of bilateral vestibular impairment (Tarnutzer et al.). For instance, patients with Wernicke's encephalopathy, show a predominantly horizontal semicircular canal impairment (Lee et al.). We also now have evidence for sequential episodes of superior vestibular neuritis leading to bilateral vestibulopathy (Yacovino et al.). Rotatory chair testing may have new applications as well, by combining gain and time constant in a new variable to help determine the severity of bilateral vestibulopathy and to track progress during treatment (Hain et al.).

Many patients with bilateral vestibulopathy suffer, and although these patients may be spared spinning vertigo and have similar rates visual height intolerance to the general population (Brandt et al.), they can be incapacitated by oscillopsia and unstable gait. Articles in this research topic show that patients with bilateral vestibulopathy depend heavily on other sensory cues such as vision and proprioception (Sprenger et al.; Medendorp et al.), and that covert saccades are triggered in order to decrease symptoms of oscillopsia (de Waele et al.). Vestibular physical therapy can be helpful. Lehnen et al. emphasize the importance of head motion while Ellis et al. propose newer cognitive interventions to improve self-motion perception.

New medical and surgical treatments are being investigated, including gene therapy to regrow hair cells and vestibular implantation. In order to determine whether new treatments are effective, well-defined and properly developed outcome measures are needed. Lucieer et al. and Anson et al. report early work toward developing new validated outcomes measures for bilateral vestibulopathy. We must also better understand the causes of bilateral vestibulopathy and establish clear diagnostic criteria. Recently the committee for the classification of vestibular disorders of the Barany society has made an important first step, defining the condition as bilateral vestibulopathy and publishing consensus diagnostic criteria (7). The manuscripts in this issue are a broad sample of the current efforts in our field to understand the pathophysiology of this disabling condition and to develop effective therapies.

## Author contributions

BW and AT jointly contributed to this editorial.

## Conflict of interest statement

The authors declare that the research was conducted in the absence of any commercial or financial relationships that could be construed as a potential conflict of interest.

## References

[1] Ford FR Walsh FB Clinical observations upon the importance of the vestibular reflexes in ocular movements. Bull Johns Hopkins Hosp. (1936) 58:0–88.

[2] Dandy WE The surgical treatment of Meniere's disease. Surg Gynecol Obstet. (1941) 72:421–25.

[3] Hobhouse E Aural vertigo. Lancet (1924) 203:821–22.

[4] Mowrer OH XXXII. Concerning the normal function of the vestibular apparatus. Ann Otol Rhinol Laryngol. (1932) 41:412–21.

[5] Dandy WE Meniere's disease: its diagnosis and a method of treatment. Arch Surg. (1928) 16:1127–52.

[6] Dandy WE Treatment of so-called pseudo-Ménière's disease. Johns Hopkins Med J. (1934) 55:232.

[7] Strupp M Kim JS Murofushi T Straumann D Jen JC Rosengren SM. Bilateral vestibulopathy: diagnostic criteria consensus document of the classification committee of the Barany Society. J Vestib Res. (2017) 27:177–89. 10.3233/VES-170619 29081426 PMC9249284

